# Anaphylaxis to hyperallergenic functional foods

**DOI:** 10.1186/1710-1492-6-33

**Published:** 2010-12-13

**Authors:** Rohan Ameratunga, See-Tarn Woon

**Affiliations:** 1 LabPlus, Auckland City Hospital, Park Rd, Grafton, Auckland, New Zealand

## Abstract

**Background:**

Food allergy can cause life threatening reactions. Currently, patients with severe food allergy are advised to avoid foods which provoke allergic reactions. This has become increasingly difficult as food proteins are being added to a broader range of consumer products.

**Patients and methods:**

Here we describe our investigations into the allergenicity of a new drink when two cow's milk allergic children suffered anaphylaxis after consuming *Wh*_*2*_*ole*^®^.

**Results:**

Our studies have shown that in comparison with cow's milk, *Wh*_*2*_*ole*^® ^contains at least three times the concentration of β-lactoglobulin. β-lactoglobulin is one of the dominant allergens in bovine milk.

**Conclusions:**

These studies have shown that modern technology allows the creation of "hyperallergenic" foods. These products have the potential to cause severe reactions in milk allergic persons. Avoiding inadvertent exposure is the shared responsibility of allergic consumers, regulatory authorities and the food industry.

## Introduction

Food allergy affects approximately 6% of children and 3-4% of adults [1]. Clinical manifestations can vary from mild abdominal discomfort to death from anaphylaxis. Currently there is no widely available specific treatment for food allergy [2]. Patients with severe food allergy are advised to avoid consuming foods to which they are allergic, in order to reduce the risk of anaphylaxis.

Avoidance of foods has however become increasingly difficult for allergic consumers. Contamination of foods with allergenic proteins can occur from harvest/production to the dinner table [3]. A further challenge for food allergic persons has been the rapid advances in food technology [4]. Proteins from a specific food can now be isolated with ease and added to another product to enhance its properties.

**Figure 1:**
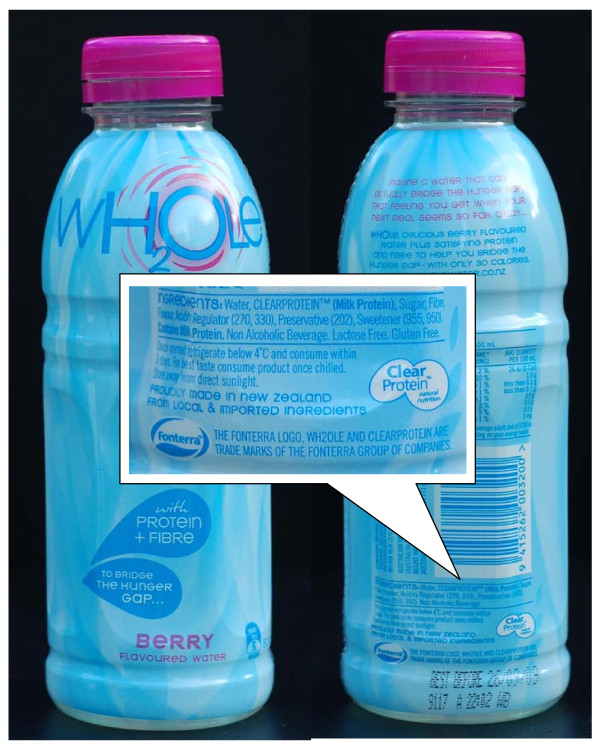
***Wh***_***2***_***ole*^® ^container. **The presence of milk protein is indicated in 3 mm letters at the rear of the container.

*Wh*_*2*_*ole*^® ^is a new drink manufactured by Fonterra of New Zealand (figure 1). *Wh*_*2*_*ole*^® ^contains high concentrations (1 g/100 ml) of bovine whey proteins, which have been added to flavoured water. The solution is a clear transparent liquid in spite of the high concentration of milk proteins. The drink is marketed as a "bridge for the hunger gap" between meals. It is placed on drinks stands in supermarkets and cafes.

Here we report the results of our investigations after two children with cow's milk allergy suffered anaphylaxis following the inadvertent consumption of *Wh*_*2*_*ole*^®^. *Wh*_*2*_*ole*^® ^contains a higher concentration of β-lactoglobulin than cow's milk and has the potential to provoke severe reactions in milk allergic persons.

## Case descriptions

### Patient 1

Patient 1 is an 18 month child. She developed urticaria after her mother consumed cow's milk and breast fed. After weaning, she had two systemic allergic reactions to cow's milk formula. On the first occasion she consumed 70 ml of formula. She developed urticaria and a hoarse voice. She then vomited. A similar reaction occurred after a second formula feed before the diagnosis of cow's milk allergy was made.

Subsequent testing for milk allergy showed a positive ImmunoCAP 7 kIU/ml (normal <0.35). She was prescribed an EpiPen^® ^Jr auto-injector (Dey Laboratories) and an anaphylaxis action plan. She was reviewed by a paediatric allergy dietician. She was placed on Neocate^® ^elemental formula. The family was very vigilant about reading food labels to avoid further milk exposure.

In June 2009 she was inadvertently given approximately 5 ml of *Wh*_*2*_*ole*^®^. Within one minute she began coughing and her voice became hoarse. She started vomiting. The family successfully deployed her EpiPen^® ^Jr and her respiratory distress improved with minutes. She was reviewed in hospital and two hours later had the outbreak of urticaria. Subsequently she made a full recovery.

Her mother observed the 5 ml of *Wh*_*2*_*ole*^® ^provoked a more severe reaction than 70 ml of milk formula. This was in spite of the reduction in the milk-specific IgE levels from 7 kIU/ml to 1 kIU/ml on ImmunoCAP at the time of the reaction to *Wh*_*2*_*ole^®^.*

### Patient 2

Patient 2 is 9 years old. She became distressed at six months of age when cow's milk formula was introduced. She had recurrent vomiting and diarrhoea. Milk allergy was diagnosed in 2000. She had a 34 kIU/ml IgE to milk in 2001. She has been carefully avoiding milk products after the diagnosis.

In May 2009 she visited a café with her parents. She selected *Wh*_*2*_*ole*^®^, which was in the drinks display cabinet. It was estimated she had approximately 5 ml of the drink. She complained of throat discomfort. There were no breathing difficulties. She then developed abdominal cramps and vomited. She did not develop urticaria. She was given antihistamines and placed under observation. She did not receive epinephrine (adrenaline). She recovered over the next few hours.

## Laboratory Methods

### Sodium dodecyl sulphate polyacrilamide gel electrophoresis (SDS-PAGE)

Samples (β-lactoglobulin [Bos d5, Sigma-Aldrich, St Louis, MO, USA], *Wh*_*2*_*ole*^® ^and trim milk: containing 1 g/100 ml fat and 3.7 g/100 ml protein) were loaded onto a 12% polyacrylamide gel and electrophoresis was performed in a Mini PROTEAN 3 cell (Bio-Rad Laboratories, CA, USA) under reducing conditions with MOPS/SDS buffer for 1 h at 150 V. Decreasing concentrations of the purified β-lactoglobulin standard were used to estimate its concentration in *Wh*_*2*_*ole*^®^. After electrophoresis, the gel was stained with Comassie Brillant Blue G-250.

### Western blotting

Western blotting was undertaken as previously described[5,6]. Following SDS-PAGE, the proteins were transferred onto a PVDF membrane in a Trans-blot Electrophoretic Transfer Cell (Bio-Rad). The membrane was blocked with 1% gelatine in blocking solution (150 mM NaCl, 5 mM EDTA, 50 mM Tris, 0.05% Triton-X) for 1 h and washed in 0.25% gelatine solution (3 × 5 min). Patient serum was diluted 1 in 10 with 0.25% gelatine solution and incubated with the membrane overnight at room temperature. The membrane was then incubated in 1:500 biotin-labelled goat anti-human IgE (Vector, Peterborough, UK) for 1 h, followed by 1:120 000 ALP-linked extravidin (Sigma) for 1 h. Following each incubation, the membrane was washed (3 × 5 min) with 0.25% gelatine solution. IgE-binding was visualised by BCIP/NBT precipitation.

### ImmunoCAP inhibition (ICI) studies

Trim milk and *Wh*_*2*_*ole*^® ^were serially diluted in 1:2 ratio with 0.9% sodium chloride. Each extract was then added to patient sera in 1:2 ratio, giving the final dilutions for the milk ImmunoCAP (1:3 to 1:100 000) and for the β-lactoglobulin ImmunoCAP (1:300 to 1:100 000). A saline and patient serum (1:2) sample was included to determine the baseline. The samples were then incubated for 1 h at room temperature and analysed on the ImmunoCAP^® ^250 system (Phadia, Uppsala, Sweden) with ImmunoCAP discs (Phadia) coated with either bovine milk (f2 CAP) or β-lactoglobulin (f77 CAP).

The response (Fluorescent units-FU) registered on the ImmunoCAP^® ^250 system for the two sets of CAPs was plotted against the dilution factors to generate the ImmunoCAP inhibition curves.

ImmunoCAP inhibition studies were not undertaken on the first patient, given the low readings (1 kIU/ml) of cow's milk IgE. This would make it difficult to interpret. ICI studies were undertaken on the second patient and another patient with high levels of cow's milk IgE. LabPlus has ethics approval for testing anonymous serum samples for quality purposes.

The study was approved by the Multi-regional Ethics Committee of the Ministry of Health in New Zealand (MEC/09/63/EXP) and the Auckland Hospital Research Office. Both families gave informed consent.

## Results

### SDS PAGE electrophoresis

**Figure 2:**
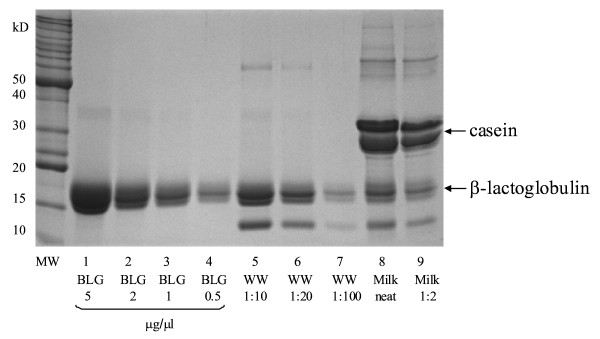
**SDS-PAGE separation of β-lactoglobulin (BLG), "*Wh***_***2***_***ole*^®^" (WW) and milk. **MW; Benchmark™ Protein Ladder (Invitrogen, Carlsbad, CA, USA); lanes 1-4: β-lactoglobulin (5, 2, 1, and 0.5 μg/μl); lanes 5-7: "*Wh*_*2*_*ole*^®^" (1:10, 1:20 and 1:100); lane 8 and 9: milk (neat and 1:2). The band below β-lactoglobulin in lanes 5-9 represents α-lactalbumin.

Commassie Blue stained SDS-PAGE of β-lactoglobulin, *Wh*_*2*_*ole*^® ^and trim milk is shown in Figure 2. *Wh*_*2*_*ole*^® ^contains higher proportion of β-lactoglobulin (lane 5 and 6, MW = 18.5 kDa) than other milk proteins. *Wh*_*2*_*ole*^® ^has a lower casein content. Faint bands for caseins can however be seen in lanes 5 and 6 (figure 2). Densitometry studies estimated *Wh*_*2*_*ole*^® ^has approximately 10 g/l of β-lactoglobulin (Figure 2), which comprises most of its stated protein content. The β-lactoglobulin concentration in *Wh*_*2*_*ole*^® ^is three times that of cow's milk [7].

### Western blotting

**Figure 3:**
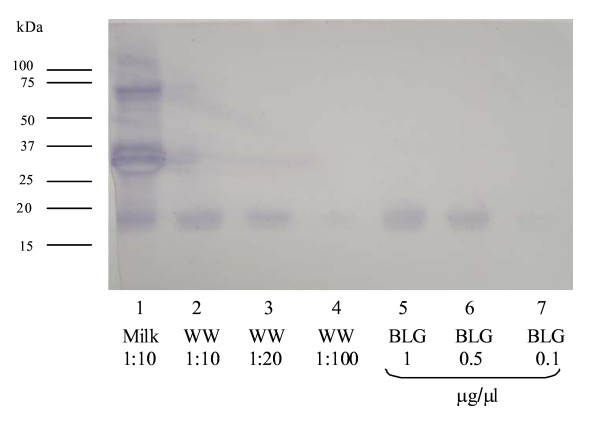
**Western blot showing specific IgE in the serum of patient 2 to milk, *Wh***_***2***_***ole*^® ^(WW) and β-lactoglobulin (BLG). **Lane 1: milk (1:10); lane 2-4: *Wh*_*2*_*ole*^® ^(1:10, 1:20, 1:100); β-lactoglobulin (1, 0.5 and 0.1 μg/μl).

Western blotting showed IgE binding in patient sera to milk proteins including caseins and β-lactoglobulin (Figure 3). Binding of IgE antibodies to *Wh*_*2*_*ole*^® ^(lanes 2-4) and β-lactoglobulin (lanes 5-7) was also confirmed.

### ImmunoCAP inhibition

**Figure 4:**
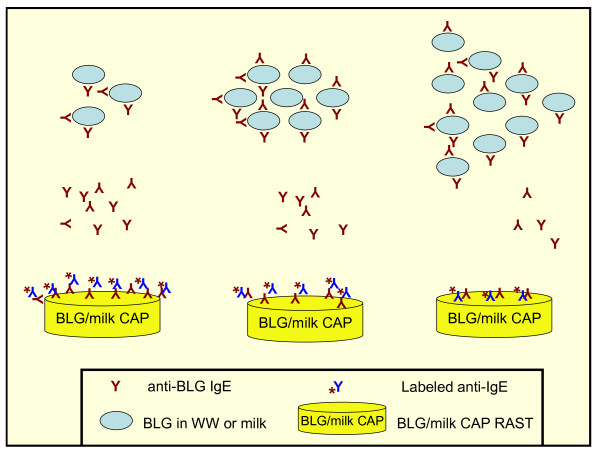
**Principle of ImmunoCAP inhibition. **As the concentration of pre incubated trim milk or *Wh*_*2*_*ole*^® ^(WW) is increased, fewer specific IgE antibodies are available to bind the RAST discs containing either β-lactoglobulin (BLG) or bovine milk.

**Figure 5:**
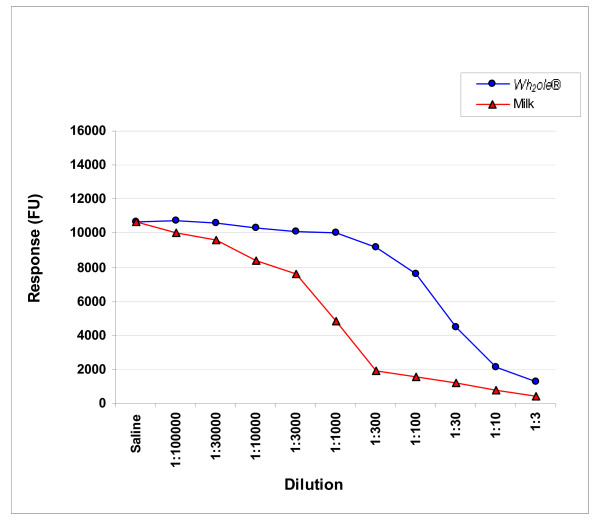
**Bovine milk ICI with trim milk and *Wh***_***2***_***ole*^®^**. FU-fluorescent units

The ICI studies were undertaken with both β-lactoglobulin and bovine milk ImmunoCAPs (figure 4). The cow's milk inhibition studies have shown pre incubation of serum with increasing concentrations of trim milk inhibits the ImmunoCAP reaction to milk ImmunoCAPs. This homologous inhibition is expected and serves as an internal control for the assay (figure 5). *Wh*_*2*_*ole*^® ^causes partial inhibition at higher concentrations (figure 5), confirming there are some caseins and other allergenic proteins in the preparation as noted in figure 2.

**Figure 6:**
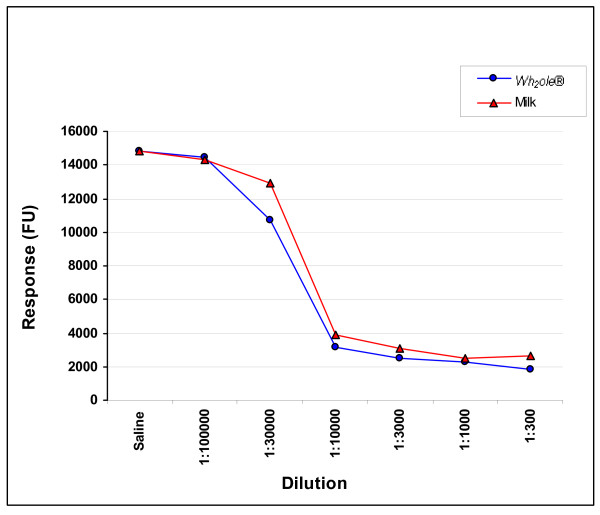
**β-lactoglobulin ICI with bovine trim milk and *Wh***_***2***_***ole*^®^**. FU-fluorescent units.

The ICI with the β-lactoglobulin caps shows inhibition of binding to the ImmunoCAP by both bovine milk as well as *Wh*_*2*_*ole*^® ^(figure 6). The inhibition curves show that *Wh*_*2*_*ole*^® ^causes more effective ICI than bovine milk confirming the higher concentration of β-lactoglobulin. This supports the results of the SDS-PAGE (figure 2) indicating that bovine milk has a lower β-lactoglobulin content than *Wh*_*2*_*ole*^®^.

## Discussion

*Wh*_*2*_*ole*^® ^is a product of modern food technology. Proteins from bovine milk have been isolated, concentrated and added to flavoured water, completely changing its appearance. This technology has been patented as "*Clearprotein*^®^" (figure 1).

*Wh*_*2*_*ole*^® ^is an example of a new class of products termed "functional foods" [8]. Health Canada (http://www.hc-sc.gc.ca) defines functional foods as products that claim health benefits beyond their nutritional value. These products are sometimes called "nutraceuticals", again reflecting their claimed health promoting properties. *Wh*_*2*_*ole*^® ^is marketed as an appetite suppressant between meals (figure 1). High concentrations of whey proteins have been shown to induce satiety [9]. Manufacturers of other functional foods claim better weight management, improved well being, reduction of diabetes risk etc [8,10].

The severity of an allergic reaction depends on several factors including the quantity of allergen consumed, the level of food-specific IgE antibodies and co-factors such as exercise. The parents of the first child observed that a much smaller amount of *Wh*_*2*_*ole*^® ^provoked a more severe clinical reaction than cow's milk formula. This was despite the decline in cow's milk specific IgE in the intervening period. This observation is consistent with our *in vitro *studies showing *Wh*_*2*_*ole*^® ^has approximately three times the concentration of β-lactoglobulin compared to bovine milk. β-lactoglobulin is the most abundant protein in bovine whey and is absent from human breast milk [7].

*Wh*_*2*_*ole*^® ^should be considered a manufactured "hyperallergenic food" as it has a higher concentration of allergenic protein compared with its food of origin. Hyperallergenic foods would also be expected to cause more severe reactions for a given weight/volume than the food of origin. Furthermore, allergic patients would be predicted to react at a lower threshold weight/volume compared with the food of origin. The ImmunoCAP inhibition studies shown in figures 5 and 6 also support our view that *Wh*_*2*_*ole*^® ^should be considered a hyperallergenic food. These foods could be considered the converse of hypoallergenic formulas where allergens have been removed or degraded to reduce the risk of allergic reactions.

We did not undertake food challenges with *Wh*_*2*_*ole*^® ^as anaphylaxis is a contraindication to such procedures. Our definition of hyperallergenic foods excludes powdered foods which can be artificially concentrated by adding less water. We have predicted the development of hyperallergenic foods by the food industry [11].

Consumption of high concentrations of β-lactoglobulin is dangerous for persons with cow's milk allergy as it is one of the dominant allergens [7]. Milk allergic patients with high concentrations of IgE antibodies to β-lactoglobulin are at particular risk from this product. Those with IgE antibodies predominantly to caseins may be at lower risk.

Until now flavoured waters sold in New Zealand have not contained protein. The original label did state *Wh*_*2*_*ole*^® ^contains cow's milk proteins in 3 mm letters on the rear of the container (figure 1). It met the food safety labelling criteria in New Zealand and Australia. (http://www.foodstandards.gov.au/thecode/). The manufacturer has subsequently changed the label of the product after becoming aware of these allergic reactions. We are not aware of any further reactions after this.

Avoidance of allergenic foods is a joint responsibility between consumers, regulatory authorities and the food industry. Food allergic patients/parents are advised to read every food label carefully, as unexpected products may contain food proteins as illustrated here. Milk is not usually associated with clear liquid. We are publishing our observations to alert consumers and physicians worldwide, that these novel products are entering the market. Allergic consumers need to be particularly vigilant as these products have the potential to cause severe reactions.

Other sources of unexpected exposure to cow's milk proteins have been described [12]. Some probiotics contain milk proteins and have triggered anaphylaxis in cow's milk allergic patients [13]. Similarly, some asthma metered dose inhalers use lactose derived from bovine milk as a stabiliser. Allergic reactions to inhalers have been described in cow's milk allergic asthmatic patients [14].

These examples illustrate the increasing difficulties food allergic consumers, regulatory authorities and the food industry will face in the coming years with advances in food technology. Food proteins are likely to be encountered in a much broader range of consumer products.

## Response from Fonterra

A prepublication copy of this paper has been supplied to Fonterra.

"Fonterra acknowledges and welcomes this research. We know that families struggle with managing food allergies and any research that heightens awareness and prevents incidence of allergic reactions is a positive outcome. In the case of Wh_2_ole^®^, the product met all New Zealand Food Safety Authority labeling requirements. Once we were alerted that people had suffered allergic reactions from it, we changed the product's packaging and worked closely with Allergy New Zealand to further alert potential allergy sufferers and the wider community about the milk protein content in the drink. Wh_2_ole^® ^was discontinued in early 2010 due to sales not meeting expectations. We have learnt from this experience and have taken steps internally to ensure we apply rigorous standards when communicating detail about functional ingredients. We remain confident in the enormous international potential of functional ingredients, such as those used in Wh_2_ole^®^, and we will continue to create innovative new products to meet consumer dietary and health requirements."

## Competing interests

The authors declare that they have no competing interests.

## Authors' contributions

RA identified allergic reactions to this product during his clinical work. He designed the experiments, sought ethics approval, and wrote the first draft of the paper. S-T W undertook most of the laboratory work described in the paper. Both authors have seen and approved the final version of this paper.
